# Evaluating Medical Image Segmentation Models Using Augmentation

**DOI:** 10.3390/tomography10120150

**Published:** 2024-12-23

**Authors:** Mattin Sayed, Sari Saba-Sadiya, Benedikt Wichtlhuber, Julia Dietz, Matthias Neitzel, Leopold Keller, Gemma Roig, Andreas M. Bucher

**Affiliations:** 1 Clinic for Radiology and Nuclear Medicine, University Hospital, Goethe University Frankfurt, Theodor-Stern-Kai 7, 60590 Frankfurt am Main, Germany; sayed@med.uni-frankfurt.de (M.S.); wichtlhuber@med.uni-frankfurt.de (B.W.); julia.dietz@em.uni-frankfurt.de (J.D.); ma.neitzel@med.uni-frankfurt.de (M.N.); keller@med.uni-frankfurt.de (L.K.); 2 Institut für Informatik, Goethe University Frankfurt, Robert-Mayer-Str 11, 60325 Frankfurt am Main, Germany; saba-sadiya@em.uni-frankfurt.de (S.S.-S.); roig@cs.uni-frankfurt.de (G.R.)

**Keywords:** medical imaging, automated segmentation, augmentation, AI, evaluation, TotalSegmentator

## Abstract

Background: Medical imagesegmentation is an essential step in both clinical and research applications, and automated segmentation models—such as TotalSegmentator—have become ubiquitous. However, robust methods for validating the accuracy of these models remain limited, and manual inspection is often necessary before the segmentation masks produced by these models can be used. Methods: To address this gap, we have developed a novel validation framework for segmentation models, leveraging data augmentation to assess model consistency. We produced segmentation masks for both the original and augmented scans, and we calculated the alignment metrics between these segmentation masks. Results: Our results demonstrate strong correlation between the segmentation quality of the original scan and the average alignment between the masks of the original and augmented CT scans. These results were further validated by supporting metrics, including the coefficient of variance and the average symmetric surface distance, indicating that agreement with augmented-scan segmentation masks is a valid proxy for segmentation quality. Conclusions: Overall, our framework offers a pipeline for evaluating segmentation performance without relying on manually labeled ground truth data, establishing a foundation for future advancements in automated medical image analysis.

## 1. Introduction

Automated medical image segmentation (AMIS) has become a well-researched field, with models covering a broad array of cases. In studies such as [1,2,3], researchers have tackled the task of building a segmentation algorithm capable of automatic segmentation of medical regions of interest (ROIs) by harnessing the power of machine learning. Common use cases are segmentation of cancerous tissue-like brain tumors [2], segmentation of specific anatomical structures, like the liver in [1], or building models capable of segmentation of multiple anatomical structures simultaneously, like the TotalSegmentator [4] or MedSAM [3]. These models show promising performance, and they are poised to support medical staff in their daily clinical workflows by automating time-consuming manual segmentation tasks, improving diagnosis accuracy, and enabling more precise treatment planning. However, little research has been done to assess and verify the accuracy of the segmentation masks generated by such models.

One of the main challenges in verifying the accuracy of AMIS models is the lack of publicly available ground truth-backed medical image data. For example, Ref. [3] found that training on only a subset of the data led to a significant drop in model performance. Moreover, even with access to a large amount of in-house data, generating ground truths remains a time-consuming process, often requiring manual segmentation of specific ROIs or a thorough review and correction of model-generated segmentation. This process becomes increasingly daunting with larger datasets or when segmenting multiple ROIs, as each ground truth would also need to be validated by at least one medical professional, in order to guarantee accuracy.

As a result, validation and verification of segmentation models are frequently conducted on unseen subsets of the training data (as in [2]) or small sets of data (as in [1]). While modern sampling and augmentation techniques can expand small datasets for validation purposes, artificially augmented images may not capture the variance and diversity often found in real-world data, which can lead to overfitting. Therefore, validation on real-world data plays an essential role in bridging the gap between experimental research and real-world application.

With these points in mind, our goal was to develop a pipeline that would facilitate a quick and accurate assessment of the quality of a segmentation mask without the need of ground truth data, regardless of the source or method of segmentation. Our intention was to predict the quality of a segmentation mask. Our pipeline achieves this through quantifying the uncertainty of model segmentation by leveraging data augmentation. In contrast to tools such as Misas [5], we do not use augmentation to assess the sensitivity of a segmentation model. Rather, we aim to quantify how well a model-generated segmentation mask fits its respective ground truth CT scan and, by extension, how much it agrees with segmentation masks produced by medical staff. The rest of this paper presents our pipeline and demonstrates its effectiveness, using the TotalSegmentator segmentation model [4]. The code to reproduce all the experiments described in this paper and supplementary data are publicly available at https://github.com/MattinSayed/Evaluating-Medical-Image-Segmentation-Models-Using-Augmentation (accessed on 16 December 2024).

## 2. Literature Review

### 2.1. Automatic Medical Image Segmentation

Medical image segmentation is a crucial yet challenging task in medical image processing, which involves the partitioning of medical scans into meaningful anatomical regions that can then be used for medical diagnostics and treatment planing [6,7]. Traditionally, this process has relied on manual annotation by medical professionals, a time-consuming and labor-intensive approach prone to inter-observer variability [8]. Over the past few decades, advancement in computational methods has led to the development of various automated tools designed to assist or entirely supersede manual segmentation [9]. Recently, deep learning techniques that adapt powerful image segmentation models—such as segment anything and feature fusion networks, which leverage convolutional neural networks (CNNs) and attention mechanisms—have been proposed [3,7]. However, ref. [9] suggested that medical image segmentation methods relying on basic image analysis (for instance, pixel/voxel similarity) or uncertainty and optimization methods (such as deep learning models) are inherently limited, and that only knowledge-based approaches that integrate domain knowledge and machine learning can attain human-like performance.

While the choice of segmentation method to be used ultimately depends on the imaging modality, specific application, and anatomical part under study [10], fully automatic and accurate medical image segmentation remains an unsolved problem [9,11], and even state-of-the-art methods require manual oversight for validation and error correction by medical professionals, particularly when segmenting small or irregular structures [12]. Therefore, developing reliable and robust frameworks for the validation and evaluation of segmentation masks remains an area of active research.

### 2.2. Segmentation Evaluation

The evaluation of automated multi-class medical image segmentation is critical for assessing model performance and comparing different approaches. Therefore, when researching this field, careful consideration is essential, to choose accurate and appropriate evaluation metrics and benchmarks [13,14].

A wide range of evaluation metrics have been used to measure segmentation quality, each suited for evaluating different aspects of model behavior. Overlap-based measures, such as the DICE Similarity Coefficient and the Jaccard Index, are most commonly used, due to their simplicity and intuitiveness. These metrics provide a measure of the overlap between predicted and ground truth segmentation, making them ideal for tasks requiring a high degree of spatial agreement. For instance, these measures are indispensable for scenarios such as tumor segmentation, where accurately capturing the entire lesion volume and excluding non-tumor regions is critical [7,14]. Volume-based metrics, such as absolute volume difference, are frequently used when clinical decisions depend on accurate volumetric measurements, such as radiotherapy planning or chemotherapy monitoring [14]. Finally, information theory-based metrics, including mutual information, and probability-based metrics like entropy are used for more nuanced evaluations, particularly in fuzzy or probabilistic segmentation [14,15]. Distance-based metrics, such as the Hausdorff distance (HD) and the average symmetric surface distance (ASSD), are critical for assessing boundary accuracy, especially in scenarios where the precise delineation of edges is clinically significant. Pair-counting metrics, though less commonly used, are valuable for analyzing segmentation consistency across datasets [14]. Despite the availability of these metrics, challenges persist in their implementation and interpretation, particularly for large datasets or segmentation involving complex anatomical structures.

To address these challenges, researchers have called for standardized and efficient evaluation tools to improve reproducibility and comparability across studies [13,14]. Specifically, Ref. [13] proposed guidelines emphasizing the importance of metric selection based on specific tasks and dataset characteristics, while [14] introduced tools for efficient and consistent evaluation. Tools such as Metrics Reloaded [16] allow researchers to map appropriate statistical metrics and image processing tasks to their research questions while also supplying researchers with information regarding the appropriate statistical metrics. Augmentations have been used to analyze the sensitivity of machine learning-based segmentation models under certain limitations, such as in [5]. While our approach also adopts augmentation, we explored and validated the use of augmentation to create a proxy for the model’s agreement with the ground truth segmentation mask rather than analyzing the sensitivity of specific models. Despite these efforts, there is currently no universally accepted evaluation framework, and the selection of appropriate metrics and methods often depends on the unique requirements of individual studies [15].

## 3. Methodology

**Figure 1:**
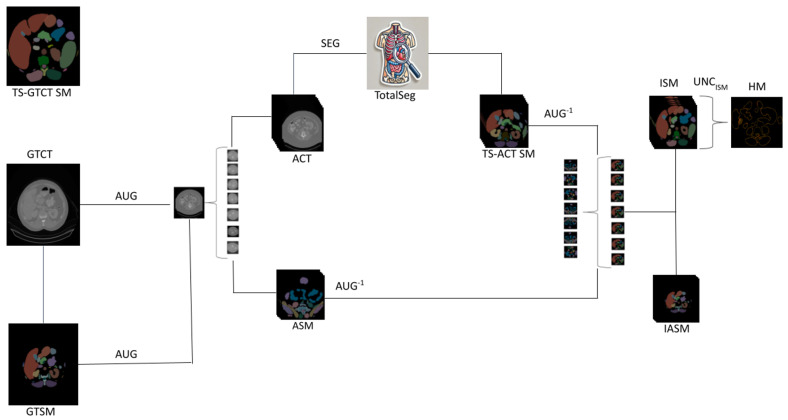
Pipeline overview (left to right): The ground truth CT scan (GTCT) and its corresponding segmentation mask (GTSM) undergo augmentation (AUG), as described in Section 3.1, to generate multiple augmented CTs (ACTs) and their corresponding segmentation masks (ASMs). Then, the TotalSegmentator (TotalSeg) segments (SEG) the augmented CT (ACT) images, to produce the TotalSegmentator augmented-CT segmentation masks (TS-ACT SMs). Next, the TS-ACT SMs and the ASMs are transformed back, using inverse augmentation (AUG^−1^), generating inverse TS-ACT SMs (ISMs) and inverse ASMs (IASMs). Finally, the ISMs are pooled, as described in Section 3.4 (UNC_ISM_), to produce the uncertainty heatmap (HM).

In this study, we used 101 CT scans (hereafter referred to as ground truth CT (GTCT)) and their corresponding ground truth segmentation mask (GTSM) from the anonymized publicly available TotalSegmentator dataset [17]. The general pipeline is visualized in Figure 1:

### 3.1. Augmentation

**Figure 2:**
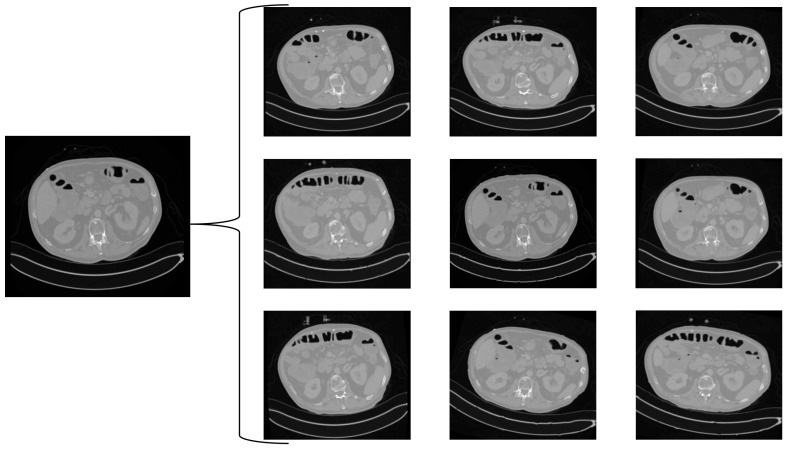
Example of augmentation: A slice of a ground truth CT (GTCT), and 9 slices from 9 different corresponding augmented CTs (ACTs).

For each CT scan, we generated 10 random augmentations, using Python version 3.12.3 [18] and torchIO [19], resulting in 10 augmented CT scans (ACTs). For an example of a GTCT scan and the corresponding ACTs, see Figure 2 below.

We defined three levels of augmentations. Level-one augmentations consisted of random rotations across all axes, where the degree of rotation for each axis was randomly sampled from a uniform distribution between −45 and 45 degrees. Level-two augmentations consisted of random elastic deformations with a control point array of (50, 50, 70) and a maximum displacement of a quarter of the voxel spacing. Level-three augmentations were made up of a level-two augmentation followed up by a level-one augmentation. Each of the 10 augmentations was equally likely to belong to any of the three levels. We applied the same augmentations to the corresponding GTSM, to obtain segmentation masks that corresponded to the ACT scans. We referred to these segmentation masks as ASMs.

It is important to note that the nnU-net [20], on which the TotalSegmentator is based, utilizes a range of data transformations during training. However, the only transformation it shares with our method is rotation. Therefore, our augmented data, which was modified with elastic deformations, was unknown to the TotalSegmentator model.

### 3.2. Segmentation

Following the augmentation step, we generated segmentation masks of the 10 ACTs as well as the GTCT for each GTCT, using the TotalSegmentator version 2.4.0, which is able to segment a maximum of 117 different ROIs [4]. For the sake of comprehension, we will refer to them as TS-GTCT SMs and TS-ACT SMs.

### 3.3. Inversion

Next, we applied the corresponding inverse augmentation to each of the 10 TS-ACT SMs, to revert them back to the affine of the GTCT. From here on, they will be referred to as ISMs.

### 3.4. Uncertainty Calculation

In order to access the uncertainty of the model, we calculated an uncertainty heatmap for each GTCT. In detail, given a set of segmentation labels $L=\{L_{1},L_{2},\cdots ,L_{n}\}$ for a specific voxel *v* across *n* segmentations, we defined the following:

1. Label Counts: We defined count(l) as the number of occurrences of each label *l* in *L*.
2. Mode and Agreement Fraction: We let $l_{{\mathrm{mode}}_{v}}$ be the label with the highest count for voxel *v*. The agreement fraction was then
  $$\mathrm{Agreement}\;{\mathrm{Fraction}}_{v}=\frac{\mathrm{count}(l_{{\mathrm{mode}}_{v}})}{n}$$

Finally, we defined the uncertainty for the voxel *v* as

$${\mathrm{Uncertainty}}_{v}=1-\mathrm{Agreement}\;{\mathrm{Fraction}}_{v}=1-\frac{\mathrm{count}(l_{{\mathrm{mode}}_{v}})}{n}$$

We did this for the n=10 ISMs per GTCT, to obtain uncertainty heatmaps for each GTCT. We provide a graphic of an example heatmap in Figure 1.

### 3.5. Statistical Analysis

We conducted the statistical analysis in Python (version 3.12.3) [18], using the numpy and pandas libraries [21,22]. In line with the recommendations of [13], and following [3], we calculated all our metrics ROI-wise and used ROI-wise DICE scores between the GTSMs, TS-GTCT SMs, and ISMs as our main metric of performance. In addition, we also calculated the averages of the aforementioned metrics for each processed CT scan file, to generate filewise metrics that allowed us to analyze the correlations between performance and the CT scan file characteristics. Specifically, we looked at file-specific metrics like voxel count and augmentation loss. Finally, we assessed the relationship of all the metrics, using Pearson correlations. In order to assess the significance of the correlations, we performed two-tailed student *t*-tests to generate *p*-values for all the correlations.

In detail, we calculated ROI-wise DICE scores for a GTSM–TS-GTCT SM and a TS-GTCT SM–ISM comparison, to assess the performance of the TotalSegmentator and its agreement with a manually labeled segmentation mask. For both comparisons, we calculated the DICE scores as follows:

For each class *c* in *C*, we defined binary masks $M_{1,c}$ and $M_{2,c}$ as

$$M_{1,c}\left(i,j,k\right)=\left\{\begin{array}{cc} 1 & \mathrm{if}\;\mathrm{voxel}\;\left(i,j,k\right)\;\mathrm{belongs}\;\mathrm{to}\;\mathrm{class}\;c\;\mathrm{in}\;M_{1},\\ 0 & \mathrm{otherwise} \end{array}\right.$$

$$M_{2,c}\left(i,j,k\right)=\left\{\begin{array}{cc} 1 & \mathrm{if}\;\mathrm{voxel}\;\left(i,j,k\right)\;\mathrm{belongs}\;\mathrm{to}\;\mathrm{class}\;c\;\mathrm{in}\;M_{2},\\ 0 & \mathrm{otherwise} \end{array}\right.$$

The DICE score for class *c* was

$$D_{c}=\frac{2\cdot \sum_{i,j,k}\left(M_{1,c}\left(i,j,k\right)\cdot M_{2,c}\left(i,j,k\right)\right)}{\sum_{i,j,k}M_{1,c}\left(i,j,k\right)+\sum_{i,j,k}M_{2,c}\left(i,j,k\right)}$$

To obtain the average DICE score across all classes:

$$\mathrm{Average}\;\mathrm{Dice}=\frac{1}{C}\sum_{c=1}^{C}D_{c}$$

The DICE scores were defined between 0 (no agreement) and 1 (full agreement).

Furthermore, we calculated the average symmetric surface distance (ASSD). Similarly to the average Hausdorff distance recommended by [13], the average symmetric surface distance aims to quantify the spatial discrepancy between two segmentation masks. Specifically, ASSD measures the average distance between the surfaces of two segmentation masks, considering both directions. Unlike the average Hausdorff distance, ASSD is less sensitive to outliers or extreme deviations, as its symmetric averaging approach provides a more reliable measure of general spacial alignment quality. We report the ASSD in millimeters (mm) of discrepancy, where higher values indicate greater discrepancy and vice versa.

As an additional metric of segmentation quality, we calculated the coefficient of variance (CV) for the DICE scores. The coefficient of variance expresses the extent of variability in relation to the mean DICE score. In the context of our study, it allowed for an assessment of consistency of segmentation performance across different ROIs or CT scans. The CV was calculated as the ratio of the standard deviation to the mean, where high values meant high variance and, therefore, low consistency, and vice versa. For clarity, we categorized the CV into three levels: low-, medium-, and high-variance. CV scores below 0.1 were considered low-variance, scores between 0.1 and 0.3 represented medium-variance, and scores equal to or higher than 0.3 were considered high-variance.

As detailed in Section 3.4, we calculated the uncertainty values for each voxel of a given GTCT, where 0 indicated the theoretical minimal average uncertainty, and where 0.9 represented the theoretical maximum average uncertainty for a given voxel. When all the ISMs assigned the same class to a voxel, there was perfect agreement (Agreement Fraction = 1), resulting in minimum uncertainty (0). When each ISM assigned a different class, there was minimal agreement (Agreement Fraction = 0.1), resulting in maximum uncertainty (0.9). In addition, we averaged the voxelwise uncertainty values across all voxels, including voxels classified as background, for each file to obtain average filewise uncertainty values.

To evaluate the impact of CT scan augmentation on performance, we measured two key metrics: augmentation loss and error rates. We calculated the augmentation loss by reversing the augmented segmentation masks (ASMs) to their original affine and comparing them with the ground truth masks (GTSMs), using the DICE scores, and then subtracting the result from 1 (ranging from 0 to 1, where 0 meant no loss). For error analysis, we identified two types of errors: Type 1 errors (ROIs present in first mask but missing in second) and Type 2 errors (ROIs appearing in second mask but missing in first). We calculated the total error rate as the percentage of all errors relative to the total possible classifications (117 ROIs × number of CT scans), ranging from 0% to 100%.

For the sake of conciseness and conformity with the guidelines presented in [13], we report ROI-wise metrics only, except for the correlations, where we report both ROI and filewise results. For each ROI-wise result, we report the mean and standard deviation, and we highlight the top two highest- and lowest-value ROIs, given the context of the analyzed metric.

## 4. Results

Out of the 101 GTCTs, we generated 309 level-one augmentation ACTs, 346 level-two augmentation ACTs, and 355 level-three augmentation ACTs. This resulted in a total of 1111 segmentation masks generated by the TotalSegmentator, of which 1010 were TS-ACT SMs and 101 were TS-GTCT SMs. After inversion, we generated a total additional 1010 ISMs and included them in the statistical analysis described in Section 3.5.

### 4.1. DICE Score Analysis

The ROI-wise DICE score analysis showed high agreement between the average DICE score across all the ROIs for the GTSM and the segmentation masks generated by the TotalSegmentator model (GTSM–TS-GTCT SM: 0.90, TS-GTCT SM–ISM: 0.92). The ROIs with the highest average DICE score were the right and left femur for the GTSM–TS-GTCT SM comparison (0.97 and 0.98, respectively) and the left upper lung lobe and the heart (0.98 for both) for the TS-GTCT SM–ISM. In contrast, the ROIs with the lowest average DICE score were the right- and left-kidney cysts for the GTSM–TS-GTCT SM comparison (0.36 and 0.37, respectively) as well as for the TS-GTCT SM–ISM comparison (0.55 and 0.64, respectively).

### 4.2. Average Symmetric Surface Distance

For the GTSM–TS-GTCT SM comparison, the average ASSD across all the ROIs was 1.19 mm with a standard deviation of 2.65 mm. The ROIs with the highest ASSD were the C4 vertebrae (27.87 mm) and the skull (7.90 mm). The ROIs with the lowest ASSD were the left and right hip (0.19 mm and 0.22 mm, respectively).

For the TS-GTCT SM–ISM comparison, the average ASSD across all ROIs was 0.65 mm with a standard deviation of 0.72 mm. The ROIs with the highest ASSD were the skull (7.28 mm) and the right-kidney cyst (3.07 mm). The ROIs with the lowest ASSD were the right hip (0.34 mm) and the second left rib (0.35 mm).

### 4.3. Coefficient of Variance

For the GTSM–TS-GTCT SM comparison, the average CV across all the ROIs was 0.19 with a standard deviation of 0.18. The ROIs with the highest CV were the left- and right-kidney cysts (1.20 for both), whereas the ROIs with the lowest CV were the left gluteus maximus (0.02) and the left clavicula (0.02); 23 ROIs showed low variation, 81 showed medium variation, and 13 showed high variation.

For the TS-GTCT SM–ISM comparison, the average CV across all the ROIs was 0.11 with a standard deviation of 0.02. Similarly to the aforementioned analysis, the ROIs with the highest CV were the left- and right-kidney cysts (0.68 and 0.64, respectively). The ROIs with the lowest CV were the aorta (0.02) and the right hip (0.02). Here, 64 ROIs showed low variation, 47 showed medium variation, and 6 showed high variation.

### 4.4. Uncertainty

The average ROI-wise uncertainty across all the ROIs was 0.05 with a standard deviation of 0.02. The ROIs with the highest uncertainty were the right and left humerus (0.13 each) as well as the right common carotid artery (0.10) and the twelfth right rib (0.10). The ROIs with the lowest average classwise uncertainty were the right and left upper lung lobes (0.01 each).

### 4.5. Augmentation Loss

The average ROI-wise loss due to augmentation across all the ROIs and augmentation levels was 0.03, with a standard deviation of 0.03. The mean loss for level-one, -two and -three augmentations was 0.04, 0.03 and 0.04, respectively. The ROIs most afflicted were the left and right humerus (0.17 and 0.16, respectively), whereas the ROIs least afflicted were the heart and the left-kidney cysts (0.004 and 0.005, respectively).

### 4.6. Distribution of Missing Classes

Across the 11,817 total instances of ROI classification that the TotalSegmentator performed on the 101 GTCTs, there were 184 misclassifications, resulting in a total error rate of 1.56%. In detail, Type 1 errors occurred 57 times, whereas Type 2 errors occurred 127 times. Looking at the frequency of misclassification per ROI, our analysis showed that the skull and the eleventh right rib were the top two for Type 1 errors, with 7 and 4 instances each. As for Type 2 errors, most misclassifications occurred in the left- and right-kidney cysts, with an instance count of 8 each.

As for the 118,170 instances of ROI classification that the TotalSegmentator performed on the ACTs, a total of 726 errors occurred (554 Type 1 errors, 172 Type 2 errors), which resulted in a total error rate of 0.61%. The ROIs with the highest Type 1 error frequency were the skull (53 instances) and the C5 vertebrae (29 instances). For Type 2 errors, the ROIs with the highest frequency were the right-kidney cyst (34 instances) and the prostate (20 instances).

### 4.7. Correlations

**Figure 3:**
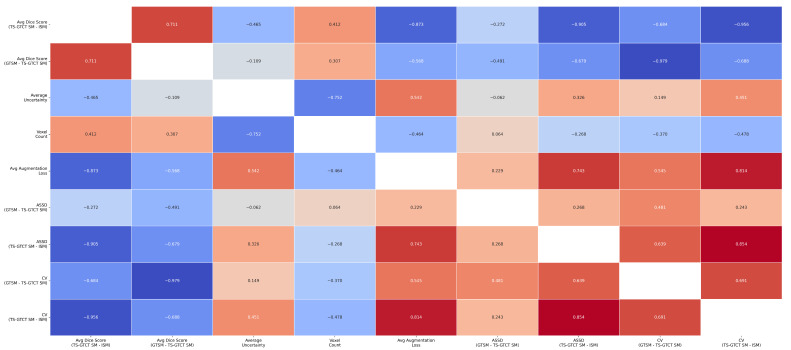
Filewise correlations: Pearson r values for all filewise metric correlations.

**Figure 4:**
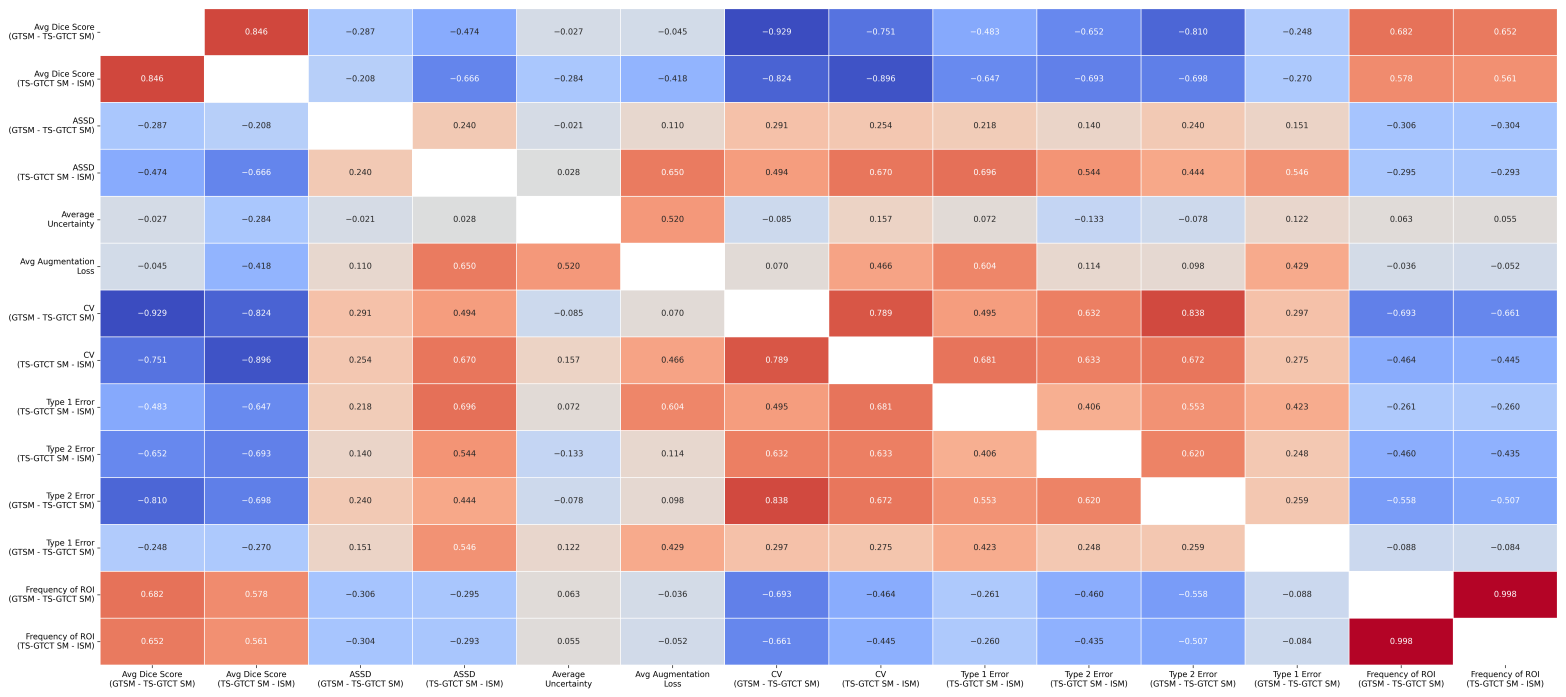
Region of Interest (ROI)-wise correlations: Pearson r values for all ROI-wise metric correlations.

Looking at the correlations in our data, we found strong positive correlations between the GTSM vs. TS-GTCT SM DICE scores and the TS-GTCT SM vs. ISM DICE scores (r = 0.85 for ROI-wise, r = 0.71 for filewise). In addition, we found several key patterns: higher DICE scores were linked to lower variability metrics, while more-frequently appearing ROIs showed better DICE scores (r = 0.68) and fewer errors. The analysis also revealed that higher voxel counts were associated with lower uncertainty (r = −0.75), lower augmentation loss (r = −0.46), and better DICE scores (r ≈ 0.31–0.41). Higher augmentation loss was connected to increased uncertainty (r = 0.52) and more Type 1 errors (r = 0.60), with uncertainty metrics showing stronger correlations in the TS-GTCT SM vs. ISM comparison. All these relationships were statistically significant (*p* < 0.01). We depict the ROI-wise and filewise correlation matrices in Figure 3 and Figure 4, respectively.

## 5. Discussion

Our evaluation revealed significant insights into segmentation model performance and validation approaches. The strong correlation between average DICE scores and various performance metrics suggests that it is possible to evaluate segmentation model performance even when sufficient manual ground truth annotation is not available, as the relationship between the TotalSegmentator-generated segmentation masks of augmented and original CT scans closely mirrors that of original CT scans segmentation masks and the manually generated ground truth segmentations. This could significantly reduce the time and resources needed for model validation. Additionally, our analysis shows that DICE scores have a stronger correlation with the coefficient of variance (CV) than with the average symmetric surface distance (ASSD), with both metrics indicating that higher DICE scores correlate with more reliable segmentation at both ROI- and filewise levels. The analysis also identified left- and right-kidney cysts as potential corner cases, exhibiting high ASSD, CV, error frequency, and the lowest DICE scores, making them valuable potential benchmark structures for evaluating automatic segmentation methods if future research confirms the aforementioned tendency.

Moreover, the study highlights the importance of dataset composition and image quality on model performance. ROI frequency significantly impacts segmentation accuracy, with more frequent ROIs showing reduced CV and improved DICE scores. This suggests that rarely occurring ROIs could be potential sources of error, emphasizing the need for balanced dataset curation in model development. Furthermore, the analysis reveals that the voxel count directly influences uncertainty estimation, with higher-resolution CT scans producing more stable uncertainty estimates. This finding has two important implications: medical staff should prioritize high-resolution imaging when clinically feasible, and developers need to ensure that their systems are robust across varying image resolutions. Additionally, our findings on data augmentation indicate that artificial variance can differ from real-world examples, highlighting the importance of modeling realistic variance during training and evaluation.

Looking at other research, we find similar tendencies to the ones found in [23]. In their paper, the authors validated a popular image segmentation deep learning model on multiple datasets and use cases, and they found that segmentation performance highly varied depending on the dataset and the task. In detail, the authors reported that image segmentation performed best when objects had clear boundaries and when given specific, unambiguous prompts. It tended to perform worse in more challenging cases, such as identifying brain tumors, where boundaries are often less distinct. Similarly to this, our study shows that the TotalSegmentator tends to fail more often in areas with a high amount of variance, whether it be natural variance of the kidney cysts or artificially introduced variance due to an unnatural patient orientation leading to poor performance for the C4 vertebrae. This is also in line with findings from [12], where the authors tested the segmentation reproducibility of the TotalSegmentator across other machine learning models by assessing volume deviation. They reported that 5 out of 34 areas showed a volume deviation of more than 5%, rendering them unreproducible. The five areas in question were the spleen, gallbladder, duodenum, and the adrenal glands. On another note, the effects of training using datasets with high variance is displayed in [24]. In that study, the authors trained a host of STU-Net-L segmentation models on multiple datasets and then validated them on the shared eight organ categories in the BTCV dataset. Their results showed equal or better DICE scores for all models when they were jointly trained on all datasets vs. being trained on a subset of them independently. Similarly, the authors of [3] found that the amount of available training data was crucial to model performance. The findings highlighted across all the aforementioned studies show the need for proper calibration of segmentation models as well as a data-centered strategy if the aim is to introduce segmentation models to daily clinical practice.

On that note, the uncertainty heatmaps generated by our model could prove useful in sensitizing models and pinpointing corner cases. As seen in [12], both the TotalSegmentator as well as an independent nnU-Net trained on the BTCV dataset showed performance dips when it came to cases with pathologic findings. This becomes more apparent when looking at the difference in performances across the functionalities of the TotalSegmentator. The authors of [25,26] reported a DICE score of 0.97 for the segmentation of anatomical structures in CT scans, 0.96 for the same task on MRI scans, and 0.75 for the segmentation of pericardial effusions on chest CT scans. Future research could evaluate the regions of high uncertainty of our heatmaps. There is reason to believe that uncertainty rises in the following cases: areas affected by pathology, areas that are naturally highly variable and in which less common ROIs are present, areas at the edges of the CT scans, areas where patient orientation is compromised, and areas afflicted by scan artifacts. Future research could focus on doing an evaluation study by, for example, letting human readers check and annotate the areas of high uncertainty, with a focus on finding key patterns. The emergence of potential key patterns would give the heatmaps more meaning, allowing them to support future segmentation models by increasing their interpretability and giving more detailed insights into how to increase performance and reliability. This is especially important in terms of future clinical applicability.

It is also worth noting that ROIs can have low ROI-wise average Dice scores in combination with low ROI-wise average augmentation loss, such as the left-kidney cyst, as described in Section 4.1 and Section 4.5. For this, it is important to consider the physical location of an ROI as well as its frequency of appearance. Taking the left-kidney cyst as an example, we know that it was located within the left kidney and that it appeared a total of six times in the ground truth segmentation mask dataset. We also know that the resolution and quality of a CT scan decreases, the further one moves away from the isocenter, which is the center of the scanner’s field of view ([27,28]), making it more susceptible to losing information that is vital to the model for segmentation. Given the relatively central position of the kidney with respect to the CT scan image borders, the below-average augmentation loss for the left kidney, and the nature of the applied augmentations, there is reason to believe that the augmentations may have had less of an effect on the segmentation mask of the kidney cyst, preserving much of their original shape. Hence, the inversion would also be less intrusive, potentially explaining the low augmentation loss as reported in Section 4.5. However, factors such as size, relatively low frequency of occurrence either during model training or during the workflow of our pipeline, distance away from the scanner’s isocenter, and inherent variance may have compromised the model’s segmentation performance. This could have resulted in inaccurate segmentation masks across the 116 instances (notably, below the average ROI appearance frequency of 790) of left-kidney cysts in the 1010 augmented CT scans. Those inaccurate segmentation masks would lead to more disagreement with ground truth segmentation masks, hence resulting in lower DICE scores, as reported in Section 4.1.

Promising results aside, this present study also had a number of limitations that should be considered. First, there was potential for bias in our evaluation metrics, since we used the same dataset that the TotalSegmentator was trained on in our pipeline assessment, due to the data’s conformity with the input requirements of the TotalSegmentator. We suspect that this may have inflated the performance results. Additionally, our pipeline was limited to testing the TotalSegmentator alone, and future work would benefit from evaluating against other segmentation tools, to prove its model-agnostic nature. The relatively small sample size of 101 CT scans, while sufficient for the initial validation, was primarily constrained by the hardware capabilities. A larger dataset that is unknown by the segmentation model in question would enable more robust statistical analysis and better representation of anatomical variations across different patient populations. These limitations suggest opportunities for future research with expanded datasets and diverse validation approaches.

## 6. Conclusions

In this study, we developed and validated a pipeline for evaluating automated segmentation models using data augmentation, demonstrating its application on the TotalSegmentator. Our statistical analysis revealed several opportunities for improvement, and the generated uncertainty heatmaps provided valuable data for further investigation. While our evaluation focused on the TotalSegmentator, the pipeline’s model-agnostic nature means it can be readily applied to other segmentation models, such as those presented in [29]. We envision our approach serving as a standardized framework for segmentation evaluation, encouraging researchers to conduct deeper analyses of their models’ behavior and performance. Ultimately, this work aims to enhance the reliability and interpretability of automated medical image segmentation systems, helping to narrow the gap between research innovations and clinical applications.

## Data Availability

The original data presented in the study are openly available on Zenodo at https://zenodo.org/records/10047292 (accessed on 16 December 2024).

## Abbreviations

AMIS: automated medical image segmentation; CT: computer tomography; ROI: medical region of interest; GTCT: ground truth computer tomography; SM: segmentation mask; GTSM: ground truth segmentation mask; ACT: augmented computer tomography; ASM: augmented segmentation mask; TS-GTCT SM: TotalSegmentator ground truth segmentation mask: TS-ACT SM: TotalSegmentator augmented computer tomography segmentation mask; ISM: inverted (augmented computer tomography) segmentation mask; HM: heatmap; ASSD: average symmetric surface distance; mm: millimeters; CV: coefficient of variance; AHD; average Hausdorff distance.
